# Pulmonary Alveolar Proteinosis Due to *Pneumocystis carinii* in Type 1 Hyper-IgM Syndrome: A Case Report

**DOI:** 10.3389/fped.2020.00264

**Published:** 2020-06-11

**Authors:** Fei Zhou Zhang, Jie Xin Yuan, Lu Qin, Lan Fang Tang

**Affiliations:** Department of Pneumology, Zhejiang University School of Medicine of Children's Hospital, Hangzhou, China

**Keywords:** pulmonary alveolar proteinosis (PAP), *Pneumocystis jirovecii* (*P. jirovecii*), hyper IgM syndrome, type 1 (HIGM1), CD40 ligand (CD40LG), infant

## Abstract

**Background:** Pulmonary alveolar proteinosis (PAP) is a rare diffuse lung disease. Reports of rare cases of PAP due to *Pneumocystis jirovecii* (*P. jirovecii*) exist in infants with immunodeficiency diseases, but no cases have been reported to date in pediatric patients with type 1 hyper-IgM syndrome (HIGM1).

**Case Presentation:** Herein, we present a case of PAP secondary to *P. jirovecii* on an infant with HIGM1. He was admitted to our unit because of cough and tachypnea. Lung biopsy confirmed the diagnosis of PAP, whereas hexamine-silver staining of the bronchoalveolar lavage fluid identified *P. jirovecii* infection. No other probable cause of PAP was observed. Whole exome sequencing indicated a novel c.511dupA (p.I171N^*^30) hemizygous mutation in the CD40 ligand (CD40LG) gene. He was cured with bronchoalveolar lavage and compound sulfamethoxazole tablets.

**Conclusions:** To our knowledge, this is the first reported case of *P. jirovecii* infection as a reversible cause of PAP in an infant with HIGM1.

## Background

Pulmonary alveolar proteinosis (PAP), first described by Rosen et al. (1) in 1958, is a rare diffuse lung disease of idiopathic etiology. Its estimated prevalence is about one in 3.7–6.9 × 10^6^ with a male/female ratio of 1:1 to 2:1 (2–5). Most patients are diagnosed between 20 and 50 years of age (2, 4–6). PAP is pathologically characterized by the presence of massive, amorphous, insoluble phospholipid-rich protein deposition in the alveolar and bronchial cavities. The first description of PAP and the vast majority of data on treatment and prognosis were connected with autoimmune PAP (1). The etiology of PAP includes surfactant-dysfunction syndromes, impaired granulocyte macrophage-colony stimulating factor signaling, hematological disorders and other malignancies, immunological diseases, infections, drugs, dust exposure, and systemic diseases (7). Etiologically, PAP is commonly classified into three types including congenital, autoimmune, and secondary PAP. Formerly, the term congenital alveolar proteinosis was used to describe surfactant dysfunction syndromes in neonates (8). Furthermore, secondary PAP primarily occurs in patients with immunodeficiency or under immunosuppressive therapy for immunodeficiency syndromes, chronic inflammatory disorders, malignancies, infections, toxic dust, and fumes (9). In 2014, Raj et al. (10) reported the first case of infant PAP secondary to *Pneumocystis jirovecii* (*P. jirovecii*) infection, and in 2016, Gallagher et al. (11) first reported a case of PAP in children with type 1 hyper-IgM syndrome but without *P. jirovecii* infection. Herein, we report *P. jirovecii* infection as a reversible cause of PAP in an infant with type 1 hyper-IgM syndrome [HIGM1, Online Mendelian Inheritance in Man (OMIM) 308230] to highlight this rare condition.

## Case Presentation

A 9-month-old boy was admitted to our unit due to dry cough and wheezing for 2 weeks, and tachypnea for 5 days. He is the second child of nonconsanguineous parents with an uneventful birth history. Medical history revealed three prior episodes of lower respiratory tract infections necessitating hospitalization and one instance of methylprednisolone treatment before referral to our hospital.

Physical examination revealed that the *Z*-score of weight for age and weight for height was 0.60; respiratory rate, 66 bpm; heart rate, 156 bpm, and temperature, 36.6°C. A depression of the suprasternal and supraclavicular fossa with coarse breath sounds without rale were noted in both lungs. The liver and spleen were non-palpable below the costal margin. Laboratory data showed a high white blood cell count, 30.61 × 10^9^ cells/L, with 41.1% neutrophils; hemoglobin, 130 g/L; hypersensitive C-reactive protein, <1 mg/L; erythrocyte sedimentation rate, 8.0 mm/h, and serum lactate dehydrogenase, 323 U/L (normal range, 135.0–215.0 U/L). Additionally, low levels of immunoglobulin A, 0.04 g/L (normal range, 0.10–0.56 g/L) and immunoglobulin G, 0.15 g/L (normal range, 3.60–9.20 g/L), and normal levels of immunoglobulin M, 0.48 g/L (normal range 0.40–1.28 g/L), were found. T cell subsets—CD19^+^, CD20^+^, CD4^+^, CD4^+^, and CD4^+^/CD8^+^–were in their normal ranges. Screening tests for HIV, syphilis, hepatitis C, mycobacteria, nocardia, Epstein-Barr virus, and fungal pathogens were negative or within normal range. Antinuclear, anti-double-stranded DNA, anti-smooth muscle actin, anti-ribonucleoprotein, and anti-neutrophil cytoplasmic antibodies were negative, respectively.

**Figure 1:**
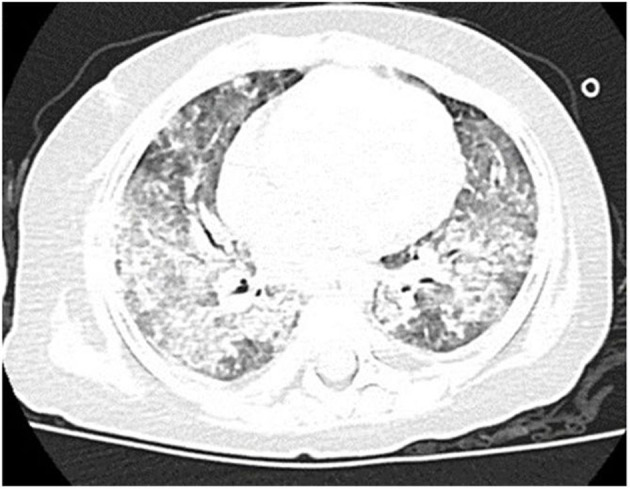
Clinical characteristics of our patient. Chest CT showed disclosed extensive progressive interstitial changes in both lungs on August 2016.

**Figure 2:**
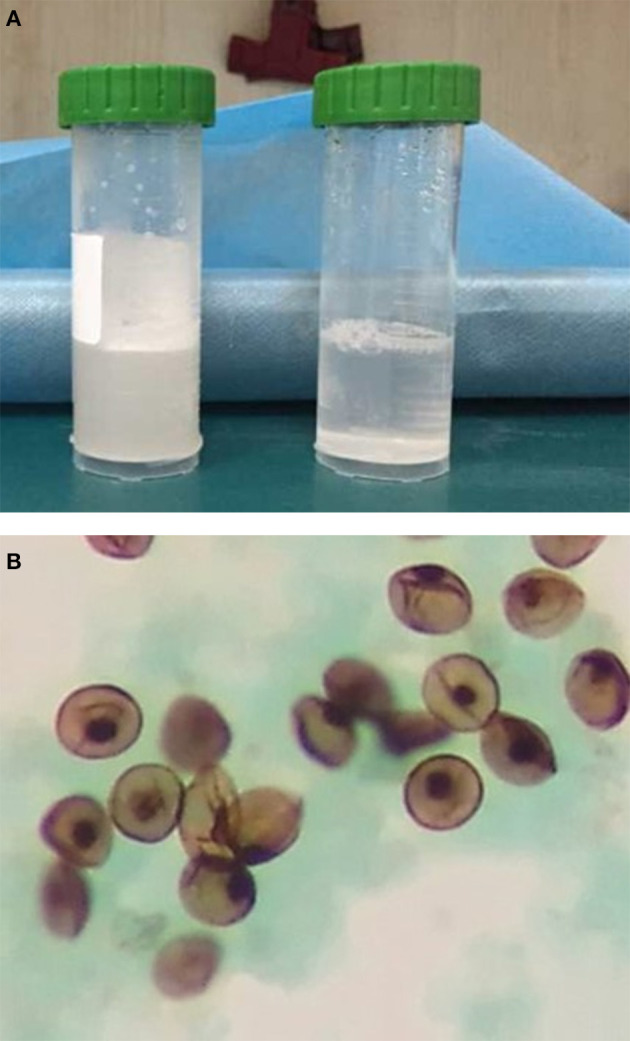
**(A)** The “milky” bronchoalveolar lavage fluid. **(B)** *P. jirovecii* was detected in the bronchoalveolar lavage fluid by silver hexamine staining.

**Figure 3:**
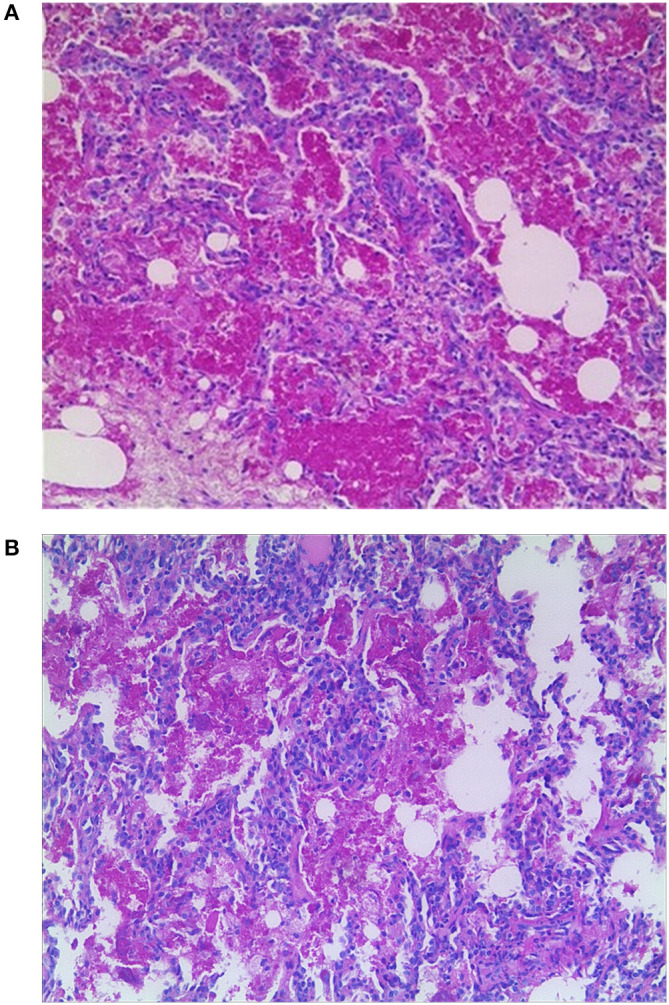
**(A)** Pathology showed large amounts of PAS-positive lipoproteins in alveolar and bronchial cavities (×100). **(B)** Pathology showed large amounts of D-PAS-positive fine granular lipoproteins in alveolar and bronchial cavities (×100).

Computed tomography (CT) scan of the chest showed ground-glass density images in both lungs (Figure 1). On the 7th day of hospitalization, bronchoscopy with bronchoalveolar lavage and lung biopsy was performed under general anesthesia. The bronchoalveolar lavage fluid appeared milky (Figure 2A), while hematoxylin-eosin staining showed extensive macrophage infiltration in the alveolar and bronchial cavities without evidence of fibrosis and hexamine-silver staining for bronchoalveolar lavage fluid revealed the presence of *P. jirovecii* (Figure 2B). Moreover, large amount of periodic acid-Schiff (PAS) stain-positive and diastase-Periodic acid-Schiff (D-PAS) stain-positive substance was found in the alveolar and bronchial cavities (Figures 3A,B), consistent with pathological characteristics of PAP and non-specific interstitial pneumonia.

Whole exome sequencing was conducted by Huada Inspection Center (Shenzhen, China) to detect gene mutation related to immunodeficiency, alveolar surface-active substance or inherited disorder associated with PAP. A c.511dupA hemizygous mutation in the EX5E region of the CD40 ligand (CD40LG, OMIM 300386) gene, present on Xq26.3 and coding for CD40L protein, was noted. Bioinformatic analysis suggested a frameshift mutation inducing the early termination of the amino acid-encoded protein (p.I171N). Genetic analysis of the patient's parents indicated maternal inheritance of the mutation.

Following the confirmation of *P. jirovecii* infection, imipenem, sulperazone, and erythromycin were administered intravenously, and sulfamethoxazole was given orally. Meanwhile, sodium bicarbonate tablets were given to alkalize urine and methylprednisolone and human immunoglobulin was used for anti-inflammatory and immune support therapy. Bronchoscopy with bronchoalveolar lavage was repeated 10 days later, and significant clearing of the lavage fluid was observed. The patient displayed gradual improvement and was eventually discharged on the 48th day of hospitalization.

**Figure 4:**
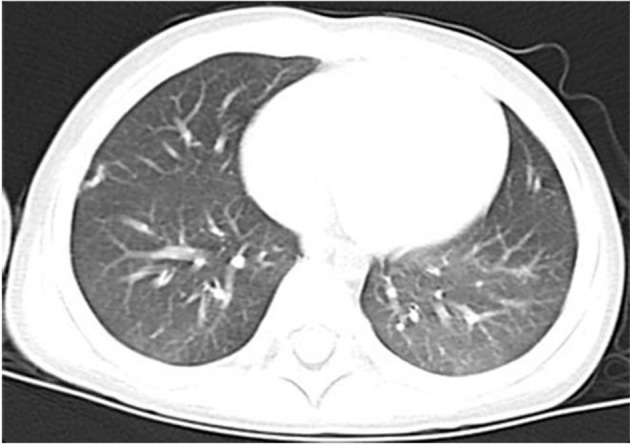
Chest CT showed significant improvement in both lungs on August 2018.

Over the course of the longitudinal follow-up, at the age of 3 years old, he was readmitted to the Department of Urology with voiding symptom persistent for more than a month. The physical examination at that time suggested that the *Z*-score of weight for age and weight for height were −0.30 and −0.40, respectively. Pelvic enhancement CT revealed a 3.5 × 3.5 × 2.5 cm calculi in the urinary bladder, subsequently extracted by cystolithotomy. The patient displayed normal growth and development since, comparable to his peers, and had no recurrence of PAP or infection during the 2-year follow-up. A chest CT showed significant improvement in both lungs in the latest follow-up (Figure 4). The parents, however, refused the proposal of hematopoietic stem cell transplantation for managing HIGM1.

## Discussion and Conclusion

PAP is an intractable interstitial lung disease involving bilateral sedimentation of lipoprotein acetous material in the lungs, affecting gas exchange and impairing the pulmonary defense against pathogenic bacteria. The diagnosis of PAP is based on clinical symptoms, imaging data, characteristics of bronchoalveolar lavage fluid, and results of lung biopsy (12, 13). The present case of PAP diagnosis was confirmed by evaluation of the bronchoalveolar lavage and pathology results. *P. jirovecii* was positively identified in the bronchoalveolar lavage fluid, although PAP secondary to *P. jirovecii* has only been reported in adults (14). Moreover, anti-*P. jirovecii* treatment is effective for treating PAP, with *P. jirovecii* being the cause of PAP in our case. Although the precise mechanism of *P. jirovecii-*induced PAP remains unclear, *P. jirovecii* should be considered as a potential etiology in pediatric cases of PAP.

*P. jirovecii* is an opportunistic pathogen, and its incidences are mainly reported in patients with immunodeficiency or under immunosuppressive therapy (15). As our case had three prior history of pneumonia with low IgA and IgG levels, immunodeficiency was considered. The patient was reported to have a heterozygous CD40LG gene mutation (c.511dupA), a frameshift mutation resulting in premature termination of the protein. CD40L dysfunction is one of the causes of rare primary immunodeficiency diseases accounting for 65–70% of HIGM1 cases (16–18). Hence, in *P. jirovecii*-infected children with or without PAP, immunodeficiency should be considered, and genetics analysis should be undertaken.

HIGM1, an X-linked immunodeficiency disorder (also named X-liked hyper IgM syndrome, XHIGM), typically presents with reduced or absent levels of IgG, IgA, and IgE, and normal to elevated levels of IgM. Clinical symptoms are complex and variable, with more than 50% of the male infants, less than a year old, developing the symptoms, whereas the figure rises to more than 90% in those younger than 4 years (19). The primary symptoms include recurrent respiratory tract bacterial infections, opportunistic infections, and recurrent or refractory diarrhea accompanied by growth retardation (20). *P. jirovecii* infection is the main cause of deaths in opportunistic pneumonia infections (21).

It remains unclear whether HIGM1 causes PAP or progresses to PAP following *P. jirovecii* infection. PAP secondary to *P. jirovecii* infection is another commonly reported variant of immunodeficiency. To our knowledge, this is the first case report of *P. jirovecii-*induced PAP in children with HIGM1. CD40L, the ligand of CD40, is a member of the tumor necrosis factor family and mainly expressed on activated CD4+ T cells (22). The CD40–CD40L interaction contributes to a majority of humoral immune responses and especially the B-cell proliferation and germinal center formation (23). Mutation in the *CD40LG* gene causes serious impairment of T-cell-dependent antibody responses because of the lack of memory B-cells and deficient induction of somatic mutations, and results in immunodeficiency with little or no circulating IgG, IgA, and IgE antibodies (24). Reports indicate that humoral immunodeficiency patients are more likely to develop opportunistic infections (e.g., *P. jirovecii*) that weaken the phagocytic function of macrophages and reduce the clearance of pulmonary surfactant, eventually leading to its overabundance, which is the most essential characteristic of PAP (7, 25, 26). Furthermore, our case displayed no recurrence of PAP during the longitudinal follow-up, supporting the opinion that HIGM1 does not directly lead to PAP.

Unlike the poor prognosis associated with congenital PAP, secondary PAP caused by infections may be reversed. However, no guideline exists for the treatment of secondary PAP in pediatric patients. Bronchial alveolar lavage is regarded as the gold standard for the effective therapy for PAP (27–29). In our case, the pulmonary symptoms resolved immediately following bronchoscopy-assisted whole lung lavage under general anesthesia. For treating *P. jirovecii* infection, we followed the European council on infections in leukemia guidelines for *P. jirovecii* pneumonia in non-HIV-infected hematology patients (30). The clinical symptoms improved after sulfamethoxazole therapy. Notably, a bladder stone was noted 3 months after discharge, which might be a renal side effect of sulfamethoxazole, although oral bicarbonate tablets to alkalize urine was administered. Therefore, alkalinity of urine and hydration should not be overlooked during sulfamethoxazole therapy. Although therapies with intravenous immunoglobulins, trimethoprim–sulfamethoxazole, and granulocyte colony-stimulating factor were reported to reduce the opportunistic infections, human leukocyte antigen-matched hematopoietic stem cell transplantation was the only radical treatment for treating HIGM1 (31, 32). However, the transplantation should be restricted to children younger than 5 years because the risk of various complications and organ damage may increase in those older than 6 years (33, 34).

To our knowledge, this is the first report of *P. carinii*-associated PAP in a HIGM1 pediatric patient. Our observations suggest that *P. jirovecii* infection should be considered as a potentially reversible cause of PAP in immunodeficient children, including infants with HIGM1.

## Data Availability Statement

All data generated or analyzed during this study are included in this published article and its supplementary information files.

## Ethics Statement

The Ethics Committee of Zhejiang University Medical College has agreed to the retrospective case report, arguing that there is no ethical dispute in the study.

## Author Contributions

LT conceptualized and designed the study, and reviewed and revised the manuscript. FZ drafted the initial manuscript. JY collected data. LQ was responsible for the follow-up. All authors approved the final manuscript as submitted and agreed to be accountable for all aspects of the work.

## Conflict of Interest

The authors declare that the research was conducted in the absence of any commercial or financial relationships that could be construed as a potential conflict of interest.

## Abbreviations

PAP: Pulmonary alveolar proteinosis
P. jirovecii: Pneumocystis jirovecii
HIGM1: type 1 hyper-IgM syndrome.
